# Substantial dust loss of bioavailable phosphorus from agricultural soils

**DOI:** 10.1038/srep24736

**Published:** 2016-04-20

**Authors:** Itzhak Katra, Avner Gross, Nitzan Swet, Smadar Tanner, Helena Krasnov, Alon Angert

**Affiliations:** 1 Ben Gurion University of the Negev, Department of Geography and Environmental Development, Be’er-Sheva, Israel; 2 The Hebrew University of Jerusalem, The Institute of Earth Sciences, Jerusalem, Israel

## Abstract

Phosphorus (P) is an essential element in terrestrial ecosystems. Knowledge on the role of dust in the biogeochemical cycling of phosphorus is very limited with no quantitative information on aeolian (by wind) P fluxes from soils. The aim of this study is to focus on P cycling via dust emissions under common land-use practices in an arid environment by integration of sample analyses and aeolian experiments. The experiments indicate significant P fluxes by PM_10_ dust due to agricultural land use. Even in a single wind-dust event at moderate velocity (7.0 m s^−1^), P flux in conventional agricultural fields can reach 1.83 kg km^−2^, that accumulates to a considerable amount per year at a regional scale. The results highlight a negative yearly balance in P content (up to hundreds kg km^−2^) in all agricultural soils, and thus more P nutrition is required to maintain efficient yield production. In grazing areas where no P nutrition is applied, the soil degradation process can lead to desertification. Emission of P from soil dust sources has significant implications for soil nutrient resources and management strategies in agricultural regions as well as for loading to the atmosphere and global biogeochemical cycles.

The dust cycle plays an important role in the Earth system^1^. Global annual dust emission is estimated to be as much as five billion tones. It is agreed that a large portion of the atmospheric dust is emitted from desert soils. However, in recent years more attention has been given to the contribution of semi-arid soils as dust sources^2^. It has been shown that agricultural activities including grazing areas and field crops can significantly accelerate disaggregation and soil loss by wind^3^,^4^,^5^. Dust emission from dry soils is controlled by surface factors, such as soil structure, vegetation, and roughness^6^, which determine the critical value (threshold) of wind (friction) velocity at which the aerodynamic drag is enough to dislodge particles from the surface and initiate their movement. The coarsening of topsoil by erosion reduces soil nutrients, leading to an eco-geomorphic feedback that reduces soil productivity and stability^7^. The essential role that dust plays in biogeochemical processes as a supplier of elements necessary for ecosystem functioning is receiving more attention^8^,^9^,^10^. For instance, it is widely accepted today that Saharan dust supplies a significant fraction of the P budget of the highly weathered soils of America’s tropical forests and of the oligotrophic water of the Atlantic Ocean, increasing the fertility of these ecosystems^11^,^12^,^13^,^14^. In contrast, increased dust emissions from cultivated sources such as agricultural soils may increase the P inputs to inland lakes or coastal oceans and accelerate eutrophication^15^,^16^,^17^. Dust particles were shown to be enriched in P^14^,^16^,^18^,^19^,^20^, probably because of their high specific surface area that contributes to high P sorption capacity. In areas where dust emission is a common phenomenon, the removal of the fine soil mineral matter due to aeolian (by wind) processes can cause substantial P losses from the soil. Such process leads to an exhaustion of the soil’s P inventory over time, which gradually decreases the fertility of agricultural and arable soils. The lost P is then redistributed globally or regionally to other ecosystems located across the dust transport pathway.

Although there is a clear association between land uses, dust emission, and related soil loss, there is no quantitative information on aeolian fluxes of P from soils. The aim of this study is to estimate the potential of aeolian P emissions from soils under different conditions of land uses and wind velocities by integration of soil-dust sample analyses and aeolian experiments. Semi-arid loess soils were examined in this study to present the phenomenon of P emission. Loess soils cover about 10% of the global land and they constitute a good representation of fertile agriculture soils that can be a dust sources (e.g. the “dust bowl” in the US).

## Results

### Soil properties and bioavailable-P

The analyses of the soil samples are presented in Table 1. The mean weight diameter (MWD) indicates the topsoil disturbance, where lower values are associated with strong disaggregation. Cultivation and grazing activities cause reduction in soil aggregate sizes due to machine operation or animal trampling^5^. Accordingly, the significantly highest MWD (p < 0.05) was found in the natural plot (N) followed by the open grazing area (G), and the cultivated soils (C and O). The long-term impacts of the land uses are reflected also in the chemical properties of the soils. The high contents of the soil cementing agents, soil organic matter (SOM), PM_10_ (including the clay fraction), and CaCO_3_ in the N plot support larger aggregates than in the other plots. The relatively high SOM content in the O plot (2.39%) is due to cropping (and higher biomass content compared to natural soil in this region), but with reduced soil tillage after the harvest that helps to keep the organic matter in the topsoil. No significant differences in major soil elements were found between the plots with typical values of element ratios (such as Ca:Mg, Al:Si) for semi-arid loess soils. In general better soil aggregation, in which more dust particles (clay, silt) are bonded to each other and held in the topsoil, enables stronger resistance for aeolian soil erosion. Assuming that higher wind threshold velocities are needed to enable transport of large-sized particles/aggregates, it is expected that the wind erosion trends will follow the soil aggregation state. The analyses of the bioavailable-P contents revealed differences between the plots (Table 1). The significantly highest value in the C plot (20.40 ppm) is related to the conventional fertilization practice. Grazing may have a positive effect on soil P, promoting nutrient cycling through livestock faeces and urine^10^. The grazing activity in the organic agricultural field (O plot) is more intensive with higher P concentration than that of the open area (G plot) due to the stubble availability but also the relatively small area in which the herd is concentrated for a longer time a year. In all the experimental soils, the content of bioavailable-P (ppm) in the PM_10_ fraction was determined (Table 2). Significantly higher values of bioavailable-P concentrations were found compared with those of the bulk samples (Table 1) that encompass the entire soil size fractions. Most of the PM_10_ fraction is composed of clays (the dominant minerals in the study area are Smectite-Montmorillonite and Illite) with specific surface area of up to 600 m^2^ g^−1^, enabling soluble phosphate ions to be adsorbed to the minerals surfaces.

### Dust emission fluxes

An example of the results obtained from the aeolian experiments is presented in Fig. 2. The results show differences in PM_10_ concentrations between the non-disturbed topsoils (Nn, Gn, Cn, On) at wind velocity of ~7 m s^−1^. The peak PM_10_ concentrations of the grazing plot (Gn) (43.2 mg m^−3^) were about 6 times higher than those in the natural plot (Nn), consistent with the aggregation results of the bulk samples in which lower MWD values were found in G plots (Table 1). The difference in PM_10_ values between the agricultural plots (O and C) was much smaller (22.5 and 27 mg m^−3^, respectively). Although the MWD values were lower in the agricultural plots compared to G or N (Table 1), the stubble remaining after the harvest and before the soil tillage enabled a better protection of the topsoil from wind erosion and thus lower PM_10_ emission rates were found in the agricultural plots than in the grazing plot (G). The measured PM_10_ concentrations (Fig. 2) were converted into PM_10_ fluxes from the soil (kg km^−2^ min^−1^) for all the experimental conditions (Table 2). In N and G plots, the PM_10_ fluxes were calculated for two wind velocities (4.5 m s^−1^ and 7.0 m s^−1^). The PM_10_ fluxes increased significantly due to the short-term surface disturbance in the natural and grazing plots (Nd and Gd). However the highest fluxes were calculated for the agricultural plots (Table 2) with mechanical tillage (Cd and Cc) and grazing intensities (Om and Os). The cultivator teeth (Cc) operate near the soil surface (8–10 cm) whereas the disk (Cd) operates in a deeper layer beneath the soil surface (10–15 cm) where it turns and mixes soil layers, thus leaving less stubble on the soil surface that protects the soil from erosion and emitting more dust^21^.

The increased grazing intensity in O plots led to higher erosion rates due to the reduction of surface cover (in this case the stubble remaining after harvest) as well as mechanical destruction of soil aggregates by animal trampling^5^. The PM_10_ fluxes of Om (721.2 μm) and Os (1253.4 μm) demonstrate the stronger negative impact of grazing in agricultural fields compared with mechanical operations, although no differences were noted in the fluxes between the control plots (On and Cn). As the soils are comparable except for land-use, in organic agricultural crop-fields, where the soil is not compacted with mechanical crusts, the grazing operation leads to disaggregation of the topsoil to form a powder-like material that is highly available for emission^5^.

### Bioavailable-P loss

The calculation of bioavailable-P loss from the soils (kg km^−2^ min^−1^) was based on the PM_10_ fluxes and the content of bioavailable-P that is held in the PM_10_ fraction (Table 2). Overall there is a positive correlation (r^2^ = 0.85) between PM_10_ and P fluxes when considering all the soil conditions (experimental plots) together. To assess the potential P loss, the contents of the bioavailable-P in the PM_10_ fraction were related to the topsoil only, which is assumed as the upper 20-cm layer of the soil that is exposed to disturbance and aeolian erosion. The results are presented in Table 2 for P loss (%) for the dry season. The aeolian erosion depends also on the presence of field crops (plots O and C) or natural vegetation (plots N and G), which can prevent dust emissions. In the study area of the northern Negev, the rain-fed agricultural fields (plots O and C) are typically covered by crops from November to May before the harvest. Thus, a realistic annual estimation of P fluxes should be based only on the dry season from June to October. The analysis of the temporal distributions of wind velocities in the dry seasons show that wind velocities of 4.5 m s^−1^ and 7 m s^−1^ occur for 739 and 151 hours in average per season, respectively (The wind data recorded in 4 meteorological stations in the studied region were analyzed for average wind velocities based on a 3-year period). A basic calculation of the potential P loss from topsoil (%) for the dry season (as per year) under wind velocity of 7 m s^−1^ is given in Table 2. It is shown that 0.58% to 60.12% of the bioavailable-P in the topsoil can be reduced due to aeolian erosion, depending on the land use and the wind velocity. Since the P concentration in the soil PM_10_ fraction (Table 2) is significantly higher than in the bulk samples (all size fractions in the soil) (Table 1), the PM_10_ fluxes in soils containing more bioavailable P (O and C) caused a relatively higher P loss compared with soils with lower concentrations of bioavailable P (N and G).

## Discussion

**Figure 1:**
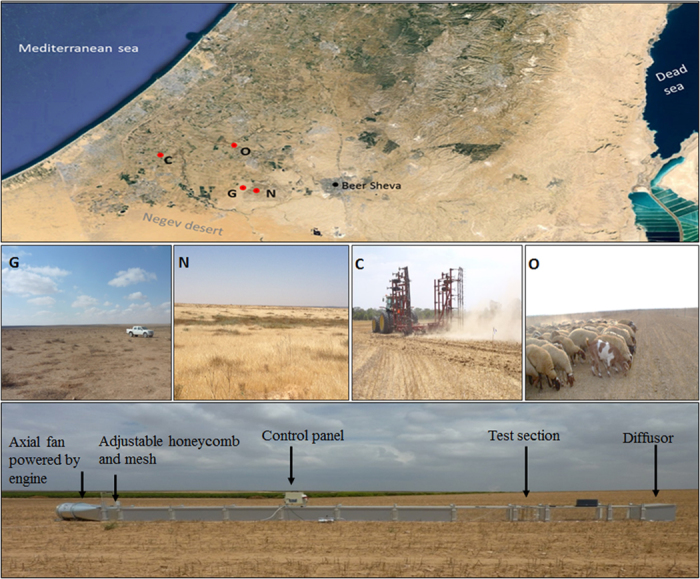
The experimental plots in typical land uses of semi-arid loess soils in the northern Negev: natural reserve (N), grazing area (G), conventional (C) and organic (O) agricultural fields. The annual average rainfall is ~200 mm. Rain events occur mainly between November and March. Winds are mainly western and can exceed 12 m s^−1^. The soil texture is mostly silt-loam (USDA). The boundary-layer wind tunnel was used for studying the dust emission (see more details in the text). The tunnel segments are presented in the air-push configuration. Instruments were installed in the test section for measuring winds and particle transport. The map produced by ArcGIS 10.0 (www.esri.com). All the photographs were taken in the northwestern Negev (Israel) by I.K.

The results above highlight how aeolian erosion leads to a significant loss of bioavailable P from the soil. In dryland systems, this process is a major agent for soil erosion over wide surfaces and thus for soil P diminution. In order to examine how aeolian erosion affects the P balance (input-output) in the soil, the inputs of P that enters the soil from external sources needs to be estimated. In this study the P loss was measured through PM_10_ fraction where the P is held in the soil. We assumed that once the PM_10_ fraction is emitted from the surface, it will be transported in the atmospheric boundary layer for a long distance from the source area^22^. Thus P lost from one field cannot be compensated for by P gained from loss from another upwind field in the study area (Fig. 1). Two major input sources of P can be identified: regional dust storms, and agricultural fertilization. The dust which settles from regional dust storms in the northern Negev originates from distal sources^23^. The annual amount of dust deposition is assumed to be equal for the all the study plots, Fig. 1) and can range^23^,^24^,^25^ from 85 g m^−2^ to 210 g m^−2^, depending on the storm intensity, with an average P content of 300 μg per 1 gram dust^26^,^27^,^28^. Considering a total dust deposition of 150 g m^−2^ (150000 kg km^−2^) during several dust storms a year, the total P deposition is 45 kg km^−2^. Thus a single dust storm in this region can contribute more than 1 kg P km^−2^, as shown also in Australia^29^.

**Figure 2:**
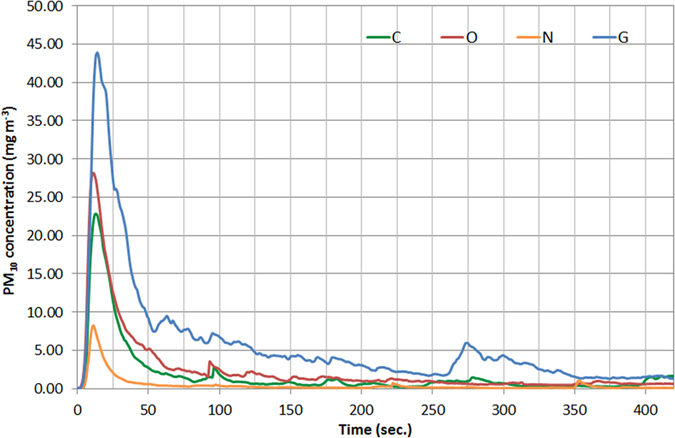
PM_10_ concentrations measured in the aeolian simulations in the experimental plots (Nn-natural, Gn-grazing, Cn- cultivation, On- organic) under wind velocity of ~7 m s^−1^. The results were used to calculate PM_10_ fluxes from the topsoil (mg m^−2^ min^−1^) (Table 2).

A typical addition of P to the soil by fertilization in the conventional agricultural fields of the northern Negev (C plot) is equal to 2000 kg km^−2^ (Ministry of Agriculture and Rural Development, Israel). Plants need P throughout their life cycle, and absorb it as orthophosphate ions (H_2_PO_4_ and HPO_4_^−^). The total P uptake in wheat fields can reach 2000 kg km^−2^, depending on the grain yield and the fertilizer treatments^30^. From a management aspect, the P uptake should be in balance with the phosphorus nutrition. In grazing areas (O and G plots), organic and inorganic P is returned to soil in faeces and urine of the livestock^31^,^32^ at an amount of ~50 kg km^−2^. A ratio between bioavailable P originated by aeolian dust deposition (Pdust) and bioavailable P emitted by aeolian processes (Ploss) can reflect the status of the soil P (Table 2). Values below 1 represent a negative balance per year. The soils of the natural reserve (N plots) received values above 1, except when a wind velocity of 7 m s^−1^ was applied after a short-term disturbance (Nn). All other soils are characterized by reduction in aeolian bioavailable-P with some considerably low values in the organic agricultural fields after grazing activities (Om and Os). This clearly shows the potential of P loss per year based on the dry season only (June-October). However there are some limitations that should be noted. First, we suggest here a topsoil-based calculation to represent the potential of P fluxes from the soil rather than actual P emission because of the complexity in collecting enough dust emission samples in space and time. The second point is particle supply in dust emission. Many topsoils are limited in the amount of dust particles that are available for transport at a specific wind velocity^5^,^22^. The resulted decrease in dust emission over time is shown in Fig. 2. It is expected that the emission rate will be lower in the next wind event if no topsoil disturbance and/or dust deposition is applied between the wind events. However most of these soils are subjected to surface disturbance over time in particular for grazing activities. In addition, the soil limitation in dust supply is a function of a specific wind (threshold) velocity^6^. Higher wind velocity will cause additional emission of dust particles that are more resistant to aeolian erosion (e.g. breakdown of larger aggregates that contain particles <10 μm). For example, amplification of wind velocity from 4.5 to 7.0 m s^−1^ in the grazing plot (Gn) enabled significantly higher PM_10_ fluxes up to 217.5 kg km^−2^ min^−1^ (Table 2). Thus stronger winds that are less frequent in the region should be also considered for the P fluxes.

**Figure 3:**
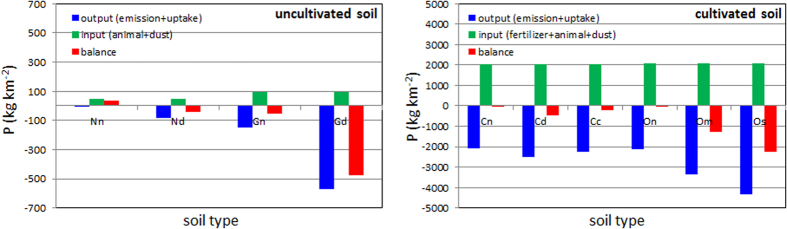
Balance of P amounts per year (kg km^−2^) in the topsoils. A–balance for natural reserve (Nn) and open grazing area (Gn) with the impact of a short-term disturbance of the topsoil (Nd and Gd). B–balance for crop fields under organic (On-no grazing, Om-medium grazing, Os-strong grazing) and conventional (Cn-no tillage, Cd-disk tillage, Cc-cultivator tillage). Note the differences in the scale values of Y axes.

Overall the results show the potential of significant reduction in P content per year in all soils under the conditions of a short-term disturbance and wind velocity of 7 m s^−1^. It was demonstrated that even in a single wind event of several minutes the soil can lose a considerable amount of P by dust emission, considering the high P concentrations in the soil PM_10_ fraction (Table 2). For example, in conventional agricultural soils with mechanical operation (Cc), the P emission can sum to 1.83 kg km^−2^ in a 5-minute wind event. Considering tens and hundreds square kilometers of such soils in the studied regions, a significant amount of P is lost and loaded into the atmosphere in a single wind event. Based on the experimental results, a yearly balance between the output and input of P in the topsoils was calculated (Fig. 3). The results clearly show a negative balance in the P content in all the soils that are subjected to agricultural activities as grazing and field crops. It can be assumed that in the conventional agricultural fields, soil fertilization by P is needed above the crop-uptake rates to maintain efficient yield production. In other land uses were no P nutrition is applied (e.g. G plots), the negative balance in P amount can indicate a soil degradation process over time towards desertification. According to our lowest estimations for harvested (crop) fields that are exposed to wind erosion, the P flux per year (dry season June-October) is ~40 kg km^−2^. At a regional scale this sums up to a significant amount of P loading to the atmosphere, especially when mechanical or grazing operations are applied in the P sources (Fig. 3). The results highlight the role of dust in the P cycle, but also the complexity in quantifying P loss from soil and its atmospheric loading. The results can reduce uncertainties in dust emission models from complex surfaces and atmospheric P transport from dust sources. The study further provides a better understanding on the soil nutrition status and the potential of P emission as well as other nutrients absorbance to soil particles (e.g., potassium, nitrogen) which is key to developing proper nutrient resource management strategies.

## Methods

### Soil tests

Samples were extracted in experimental plots that represent major land uses in the northwestern Negev region (Fig. 1). The grazing plots (G) are characterized mainly by bare soils with patches of dwarf shrubs, sparse herbaceous cover, and seasonal biological crust. The natural plots (N) are situated within a closed area (natural reserve) without any human interference during the last decades. These plots are characterized by coverage of biological crust, as well as annual and seasonal vegetation. Samples in the agricultural fields were related to the fallow phase of a rain-fed winter cereal–summer fallow crop rotation which is a major agricultural practice in many places throughout the world. Two systems were studied (Fig. 1): conventional agriculture practice (C) and organic agriculture practice (O). The first practice is the most common one in the study area and includes the use of pesticides and chemical fertilization. After harvesting the winter crops, mechanical tillage is performed before sowing the following crop. The organic practice is characterized by reduced tillage techniques and by avoiding the use of pesticides and chemical fertilization. After harvesting the winter crop and before sowing the following crop, the stubble is grazed by herds of sheep and goats. All soil samples were taken during the dry season from the topsoil layer. A total of 72 bulk samples were analyzed (4 land uses, 18 replicas). Laboratory tests were performed for physicochemical characterization of the soil samples, including Aggregate Size Distribution (ASD), Particle Size Distribution (PSD), Soil Organic Matter (SOM), Carbonates (CaCO_3_), and elemental composition. The samples were analyzed as follows. ASD was derived from the bulk samples using the dry sieving method at the fraction diameters (μm) of 63, 125, 250, 500, 1000, and 2000 using an electronic sieving system (RETSCH AS 300 Control). Each size fraction was weighted separately to calculate the mean weight diameter (MWD) of the sample. PSD was derived for all the bulk samples (N, G, O, C). A laser diffractometer (ANALYSETTE 22 MicroTec Plus, Fritsch) was used to measure the particles in the size range of 0.08–2000 μm. Replicates (100 mg) of each sample were dispersed in Na-hexametaphosphate solution (0.5%) and by sonication (38 kHz). PSD data was calculated using the Fraunhofer diffraction model with a size resolution of 1 μm using MasControl software. SOM content (%) was determined by the dry combustion method^33^. A 5 g sample of crushed oven-dried (105 °C for 24 h) soil was placed in a combusting oven at 375 °C for 17 h. CaCO_3_ was determined as mass content (%) by the Calcimeter device. The carbonates present in a sample are converted into CO_2_ by adding hydrochloric acid 8% (HCl). The calcium carbonate content can be calculated with reference to a standard sample of analytical (100%) CaCO_3_. Elemental composition was obtained by the X-Ray Fluorescence (XRF) method using an XRF spectrometer, PANalytical Co., model Axios (wavelength dispersive -WDXRF, 1kW). The measurement was conducted on 1 g of air dried powdered soil. Omnian software was used for quantitative analysis. All the soil data were statistically analyzed with SPSS for means and significance at p ≤ 0.05.

### Phosphate analysis

P concentrations were determined for the bulk samples (N, G, O, C) and for the size fraction of the fine particles (<10 μm) that are in the bulk samples. The fine fraction from each sample was derived by the dry sieving method (Gilsonic AutoSevier GA-6, Gilson inc, Ohio, US). We measured the concentrations of the resin-extractable P fraction (resin-P), which is considered analogous to the P that is available to biological uptake^34^,^35^. To extract the resin-P from the bulk sample and the fine fraction, a subsample of 0.5 g (10 mg for the fine fraction) was shaken on an orbital shaker with anion exchange resin membranes (BDH-55164) in 50 ml (10 ml) of double deionized water for 24 hours. To remove phosphate from the membranes, the resin membranes were shaken overnight in 5 ml of 0.2 M HNO_3_^14^,^36^. Phosphate concentrations were determined by molybdate colorimetry^37^. The average difference between duplicate samples was 1.3%.

### Aeolian experiments

Aeolian experiments in the field were conducted in the experimental plots with a boundary layer wind tunnel (Fig. 1). Boundary-layer wind tunnels enable aeolian simulations under standardized quasi-natural wind conditions^38^ and provide quantitative information on aeolian particle transport including sand fluxes^39^ and dust emission rates from soils^3^,^4^,^40^,^41^. The wind tunnel has a cross sectional area in the order of 0.5 × 0.5 m with open-floored working sections of up to 10 m length^5^,^22^. Air push or air suction flow in the tunnel is generated by an axial fan up to a maximum velocity of 18 m s^−1^. Instruments installed in the test section of the tunnel enable quantification of: wind profile for the calculation of frictional velocity and roughness height, samples of aeolian sediments, sand fluxes, and dust concentrations including PM_10_. The wind tunnel was operated on bare soil surfaces in all plots. The experimental conditions for plots N and G included two soil treatments, natural state (Nn and Gn) and disturbed surface (Nd and Gd). The soils were treated in the field to simulate a short-term disturbance of human activities that is common in semi-arid soils. The topsoils were artificially disturbed by mechanical operation, thus Nd and Gd represent un-crusted topsoil and reduced soil aggregation compared with Nn and Gn soils, respectively. The tunnel fan was run at two frequencies (32 and 41 Hz), representing medium wind velocity in the study area which is above the threshold of particle transport (~4.5 m s^−1^), and a higher wind velocity which represents typical aeolian erosion conditions in the studied area (~7.0 m s^−1^). Each test above was conducted in three field replicates (a total of 24 aeolian experiments). Different soil treatments were studied in the agricultural plots. Soil treatments in the conventional practice included plots of no-till (Cn), disk-tillage (Cd) (12–15 cm tillage depth) and cultivator-tillage (Cc) (8–10 cm tillage depth). The organic practice included plots of no grazing (On), medium grazing (Om), and strong grazing (Os). The tests in the agricultural plots were conducted under erosional conditions (41 Hz). Each test above was conducted in three field replicates (a total of 18 aeolian experiments). The PM monitor installed in the test section (DustTrak, TSI) (Fig. 1) enabled recording PM concentration (μg m^−3^) at intervals of 1 second. Each experiment was performed for 420 seconds. The recorded PM_10_ data were converted into fluxes from the soil surface (mg m^−2^ s^−1^) based on the dimensions of the wind tunnel and the volume of the air flow.

## Additional Information

**How to cite this article**: Katra, I. *et al.* Substantial dust loss of bioavailable phosphorus from agricultural soils. *Sci. Rep.* **6**, 24736; doi: 10.1038/srep24736 (2016).

## Figures and Tables

**Table 1:** Mean values of topsoil properties in the bulk samples of the experimental plots (N-natural, G-grazing, C- cultivation, O- organic): bioavailable-P content, percentages of particulate matter >10 μm (PM_10_), soil organic matter (SOM), carbonates (CaCO_3_), and major elements (Ca, Mg, Na, Al, Si, K, F), and mean weight diameter (MWD) of soil aggregates.

Site	MWD (μm)	P (ppm)	PM_10_ (%)	SOM (%)	CaCO_3_ (%)	Ca (%)	Mg (%)	Na (%)	Al (%)	Si (%)	K (%)	Fe (%)
N	2531	5.40	41.87	2.28	22.42	22.41	2.53	2.02	6.46	26.75	3.47	6.63
G	1033	6.30	30.66	1.90	12.13	14.61	2.96	2.60	7.08	32.63	4.07	5.99
O	860	9.70	29.10	2.39	12.13	11.15	2.63	3.43	8.66	31.60	4.39	6.77
C	590	20.40	31.20	1.54	7.98	12.66	3.12	2.28	8.17	29.88	4.06	6.79

**Table 2:** Dust emission from the topsoil (0–20 cm) under different wind velocities in N (natural) and G (grazing) sites with undisturbed (Nn and Gn) and disturbed (Nd and Gd) surface conditions, and in O (organic) and C (cultivation) sites with no till (On and Cn) and disturbance of medium grazing (Om), strong grazing (Os), disk-tillage (Cd), and cultivator-tillage (Cc).

Wind velocity (m s^−1^)	Site	Sample	P in PM_10_ fraction (ppm)	PM_10_ flux (kg km^−2^ min^−1^)	P flux (kg km^−2^ min^−1^)	Bioavailable-P in topsoil (kg km^−2^)	P loss topsoil (%)	Ratio Pdust/Ploss
4.5	N	Nn	58.2	2.9	0.0002	1296	0.58	6.01
		Nd		14.26	0.0008		2.84	1.22
	G	Gn	95.4	24.54	0.0023	1512	6.86	0.43
		Gd		70.52	0.0064		19.72	0.15
7.0	N	Nn	58.2	17.6	0.0010	1296	0.72	4.84
		Nd		159.7	0.0093		6.51	0.53
	G	Gn	95.4	217.5	0.0208	1512	12.46	0.24
		Gd		825.8	0.0788		47.30	0.06
	O	On	232.3	74.4	0.0193	4896	3.57	0.26
		Om		721.2	0.1866		34.59	0.03
		Os		1253.4	0.3243		60.12	0.02
	C	Cn	258.7	54.6	0.0127	2328	4.95	0.39
		Cd		311.4	0.0723		28.21	0.04
		Cc		158.4	0.3678		14.35	0.13

The P flux was calculated based on the basis of bioavailable-P content (ppm) within the PM_10_ fraction of the soil (Table 1). The P loss (%) from topsoil (upper 20-cm layer of the soil) per season was derived according to the number of hours (151) of wind velocity 7 m s^−1^ in the study region during the dry season (June-October) when the soil is exposed to wind erosion. Pdust/Ploss is the ratio between the yearly amounts of natural P input on a square kilometer by aeolian dust deposition in the region (Pdust) (150000 kg km^−2^ per year) and P loss by aeolian erosion during the dry season–values below 1 represent negative balances.
